# The Economic Burden of Meningitis to Households in Kassena-Nankana District of Northern Ghana

**DOI:** 10.1371/journal.pone.0079880

**Published:** 2013-11-21

**Authors:** Patricia Akweongo, Maxwell A. Dalaba, Mary H. Hayden, Timothy Awine, Gertrude N. Nyaaba, Dominic Anaseba, Abraham Hodgson, Abdulai A. Forgor, Rajul Pandya

**Affiliations:** 1 School of Public Health, University of Ghana, Accra, Ghana; 2 Navrongo Health Research Centre, Ghana Health Service, Navrongo, Ghana; 3 National Center for Atmospheric Research, Boulder, Colorado, United States of America; 4 Research and Development Division, Ghana Health Service, Accra, Ghana; 5 War Memorial Hospital, Ghana Health Services, Navrongo, Ghana; Fondazione Bruno Kessler, Italy

## Abstract

**Objective:**

To estimate the direct and indirect costs of meningitis to households in the Kassena-Nankana District of Ghana.

**Methods:**

A Cost of illness (COI) survey was conducted between 2010 and 2011. The COI was computed from a retrospective review of 80 meningitis cases answers to questions about direct medical costs, direct non-medical costs incurred and productivity losses due to recent meningitis incident.

**Results:**

The average direct and indirect costs of treating meningitis in the district was GH¢152.55 (US$101.7) per household. This is equivalent to about two months minimum wage earned by Ghanaians in unskilled paid jobs in 2009. Households lost 29 days of work per meningitis case and thus those in minimum wage paid jobs lost a monthly minimum wage of GH¢76.85 (US$51.23) due to the illness. Patients who were insured spent an average of GH¢38.5 (US$25.67) in direct medical costs whiles the uninsured patients spent as much as GH¢177.9 (US$118.6) per case. Patients with sequelae incurred additional costs of GH¢22.63 (US$15.08) per case. The least poor were more exposed to meningitis than the poorest.

**Conclusion:**

Meningitis is a debilitating but preventable disease that affects people living in the Sahel and in poorer conditions. The cost of meningitis treatment may further lead to impoverishment for these households. Widespread mass vaccination will save households' an equivalent of GH¢175.18 (US$117) and impairment due to meningitis.

## Introduction

While epidemics of meningitis occur throughout the world, the greatest burden of the disease is in the “meningitis belt” of the Sahel of Africa, where widespread epidemics occur about once a decade, but not predictably.

In addition to a steadily invariant fatality rate of 10%, about 10% of cases result in sequelae [1], both of which have long-term economic impacts on the households of those afflicted. Treatment often results in significant health care costs to the households. These factors suggest that meningitis poses a major risk to livelihoods in resource poor settings [2] like the Sahel.

Costs of illness studies, related to a variety of diseases including meningitis, have reported that impoverishment results when households are forced to spend 40% of their non-food expenditures on medical expenses [3], [4]. Even much smaller expenditures on health care have been reported to be financially disastrous to households [5]. In some cases, households coping strategies include selling their assets or borrowing from friends and relatives, which may exacerbate the initial financial impact and trap them in poverty [5].

Although there is growing knowledge of the proportion household expenditure spent on health [6], little is known either about the affordability of care or the loss of income due to meningitis or the extent to which meningitis contributes to household impoverishment. The objective of this study is to estimate the direct and indirect costs of meningitis to households in northern Ghana using the COI approach, as a first step in quantifying the economic impact of meningitis across the Sahel.

Cost of illness (COI) studies are usually analyzed either from the societal perspective (i.e. including the patient and the health system/provider costs), health system/provider perspective (i.e. including costs incurred by the health provider) and patient perspective (i.e. the patient only or patient and care giver costs) [7]–[9]. This study adopted a patient perspective, focusing on costs incurred by the household of the individual with meningitis. Four broad categories of costs; direct medical costs, direct non-medical costs, indirect costs and intangible costs are usually undertaken in COI studies [7], [8]. Direct medical costs include drugs, laboratory tests, and consultation fees. Direct non-medical costs relate to transportation costs, food and lodging that result directly from the illness. Productivity losses often referred to as indirect costs are costs associated with lost earnings due to impaired ability to work or to engage in leisure activities attributable to the illness. Productivity losses are often estimated using the human capital approach. Human Capital Approach (HCA) assumes that the productivity losses associated with a worker who stops work due to illness or death are the average annual wage for their age and gender from the time that the worker stops work until the age of retirement. HCA is estimated by multiplying the cumulative number of missed workdays by a daily wage [10], [11]. Productivity losses associated with meningitis were estimated as the income lost due to meningitis morbidity using the number of workdays lost. Data on intangible costs that result from pain, discomfort, quality of life associated with the disease were not collected for this study.

## Materials and Methods

### Ethics Statement

The interviews were conducted in person and in the local language most comfortable to the interviewee, by trained staff from the Navrongo Health Research Centre, using a structured questionnaire. Written informed consent was obtained from all adult participants. For children less than 18 years of age, written informed consent was obtained from an adult caregiver who also answered survey questions. Ethical clearance for the study was obtained from the Navrongo Health Research Centre Institutional Review Board (Clearance number: NHRCIRB091).

### Study site

The study was conducted in the Kassena-Nankana district of northern Ghana. The district is primarily rural with only 10% of its residents living in the district capital of Navrongo. It covers an area of about of about 1675 km^2^ and has a population of 152,000 people [12]. The Kassena-Nankana district population settlement pattern is characterized by extended families living in dispersed compounds surrounded by farmlands.

Descent among Kassena-Nankana is patrilineal and usually two or more nuclear families come together to form a compound. The compound head is responsible for the social, religious, economic and political wellbeing of all the compound members. Decision-making, including decisions about health-care, is generally hierarchical. With the dispersed settlement pattern and few compact villages, health service delivery is often difficult.

The district reflects ecological conditions characteristic of the Sahel with a semi-arid Guinea savanna with one rainy season from June through October. The district is thus caught within the meningitis belt, and outbreaks are not uncommon. Although most outbreaks in the district are caused by *Neisseria meningitidis* with Group A being the commonest type, there have also been cases of *Streptococcus pneumonia* and *Haemophilus influenzae* type b [13]. A major outbreak between 1996 and 1997 in the Kassena-Nankana district recorded 1396 cases with 65 deaths [14]. Meningitis cases recorded in the district in 2002, 2003 and 2004 were 215, 185 and 216 cases respectively. The total meningitis cases in the Upper East region which includes Kassena-Nankana and 8 other districts from 2007 to 2010 was 1,080 cases with 167 deaths [15]. Comparatively the Kassena-Nankana district had more cases (253) of meningitis of than the eight districts of the region. The Ghana Health Service organizes free yearly national mass vaccinations against meningitis for adults and children. In the past, polysaccharide vaccines have been used to manage meningitis in Ghana. These vaccines were protective for three years. In 2012, Ghana introduced meningococcal A conjugate (MenAfriVac) vaccine in the three northern regions that are caught within the meningitis belt. The mass vaccination campaign in these regions target people aged between 1 and 29 years. The MenAfriVac has several advantages over the polysaccharide vaccines. The vaccine reduces the carriage of the bacteria in the throat, it is expected to confer long-term protection not only for those who receive the vaccine but also others who would otherwise have been exposed to meningitis, and it is expected to be particularly effective in protecting children less than two years who do not respond to polysaccharide vaccines [16]. Ghana Health Service follows three main strategies proposed by WHO to deal with meningitis in the country- epidemic preparedness, prevention and response. The preparedness focuses on keen surveillance comprising case detection, investigation and laboratory confirmation; prevention involves vaccinating the population; whilst response consists of prompt and appropriate case management. The district meningitis vaccination coverage during the 2012 MenAfriVac campaign was 78.1% [17] which is below the 90% target. There has been an upward trend in vaccinations coverage in Ghana in the past 10 years. The national coverage of vaccinations ranges between 75% and 89% [18], [19].

The district health services include a district hospital, six government-run health centres, one private clinic, two mission clinics the Navrongo Health Research Centre laboratory, several private chemists, a number of traditional healers, traditional birth attendants, and several itinerant drug vendors. With the dispersed settlement pattern and few compact villages, health service delivery is often difficult.

The KND is a Health and Demographic Surveillance System (HDSS) site of the Navrongo Health Research Centre. As part of the Navrongo HDSS (NHDSS) activities, households are visited three times a year to collect data on pregnancies, births, morbidity, deaths, migration, marriages, vaccination coverage and other vital socio-economic variables [20].

### Data collection

A cost of illness (COI) survey was conducted between May 2010 and May 2011 with 80 households with confirmed meningitis cases. This COI analysis was done among cases from a case-control study which aimed to determine the socio-economic impacts of meningitis in the KND towards a goal of better understanding environmental determinants of meningitis and impacts and costs associated with meningitis morbidity/mortality in Ghana [21].

All meningitis cases that occurred during 2008, 2009 and until September 2010 were obtained from the District Health Management Team (DHMT) of the Kassena-Nankana District and the Navrongo Health Research Centre laboratory. Of the 189 confirmed cases that were obtained initially, 55 died before their homes were visited, 32 had migrated from the district, 17 were untraceable, and 5 denied having contracted meningitis. Thus only 80 cases were interviewed. The high number of deaths among the confirmed cases (55) is not surprising as the case-fatality rate for pneumococcal meningitis in the district has been reported at 44.4% [13].

Direct and indirect costs covered costs associated with care at health facilities and any other treatment source. Interviewees were asked to estimate direct medical expenditure on drugs, consultation fees and hospitalization costs. Direct non-medical cost included cost of transportation, lodging and meals incurred during treatment and recovery. Indirect costs questions covered time spent caring for a meningitis case and the subsequent loss of income or productivity resulting from it. Where there was productivity forgone but no lost income, the opportunity costs of looking after a sick child or adult were estimated using a minimum wage. Sequelae was defined as after effects of meningitis that occur sometime later after patients have been treated and discharged. For sequelae, only direct costs of treatment were obtained but productivity losses of patients and caregivers were not explored for sequelae. The Knowledge, Attitude and Practice survey also collected data on age, sex, income, assets and possessions of the households that participated in the COI survey.

### Data processing and analysis

Data were doubled-entered using Epidata 3.1 and analyzed using STATA 11.0^©^. Costs of treatment were estimated by household. The sum of direct medical costs and indirect medical costs were summed and divided by number of households to arrive at the total and average costs per treatment. The number of days lost during the meningitis episode was estimated and then multiplied by the minimum wage to arrive at the total indirect costs per case.

Data for assessing the socioeconomic status of all households is collected through the Navrongo Health and Demographic Health Surveillance System [20]. This database has information on all households in the district. We linked the study participants to the NHDSS household socio-economic data to generate the household wealth index using Principal component analysis technique. The PCA involves a mathematical procedure that transforms a number of correlated variables into a smaller number of uncorrelated variables, thus allowing variables that are collinear to be grouped together to form a composite index [22], [23]. In this study therefore, wealth or SES of the household was measured in terms of assets and possessions using PCA. The assets and possessions used for the construction of the wealth index were type of material for wall, roofing material, cooking utensils, toilet facility, source of drinking water and cooking fuel. Household possessions included bicycle, motorbike, car, radio, bed, sewing machine, tape player, TV, DVD, mobile phone, refrigerator, cattle, sheep, goat, pig and donkey. Households were then assigned to five quintiles as used in other studies like the Ghana Demographic Health Surveys (GDHS) [24]–[26]. These quintiles described wealth levels and represented as: least poor, less poor, poor, very poor, and poorest.

All costs were captured in Ghana Cedis and then converted during the analysis to US$ using the average exchange rate of 1 US dollar to 1.5 Ghana Cedi for 2009.

## Results

### Socio demographic characteristics of meningitis cases

Costs of treating meningitis were obtained for 80 cases that completed the interview. There were more females (44) with meningitis than males (36). Fifty-nine percent of cases were under 18 years of age. Majority of cases reported no education 62.5% (50) and 26.3% (21) reported only primary education. Majority 90% (72) of the cases worked in the informal sector. Twenty-four cases belong to least poor quintile and 6 cases to the poorest quintile (Table 1).

**Table 1:** Socio demographic Characteristics of Meningitis Cases.

Category	Subcategory	Cases n = 80	Percent (%)
**Sex**	Male	36	45
	female	44	55
**Age**	Below 18yrs	47	58.8
	Above 18yrs	33	41.3
**Educational level**	No education	50	62.5
	Primary/JHS	21	26.3
	SHS	5	6.3
	Tertiary	4	5
**Occupation**	Unemployed	12	15
	Self employed	60	75
	Government employed	8	10
**Wealth quintile**	Least poor	24	32
	Less poor	11	20.6
	Poor	12	16
	Very poor	22	29.3
	Poorest	6	8.0
**Vaccination**	Vaccination	50	62.5

### Household Health Seeking for Meningitis

Of the 80 cases of confirmed meningitis, 62.5% (50) reported that they had been previously vaccinated. Seventy percent (54) of the cases sought care from a clinic, health worker, health center or hospital while 19. 5% (15) sought care from the drug shop and 10.6% (8) from a traditional healer.

### Costs of Treating Meningitis

Of the 80 cases, 77 cases provided cost of illness information. Households spent GH¢75.70 (US$50.47) on average in direct costs per case of meningitis. The average direct medical cost arising from consultation, drugs and laboratory services was GH¢124.6(US$83.07) per case. Of this, households spent on average GH¢77.3 (US$51.5) per case in a hospital, and additional GH¢ 25.5(US$17) for traditional care, GH¢17.4(US$11.6) at the clinic, GH¢3.3(US$2.2) at a drug shop and GH¢1.4(US$0.93) at a community health worker. Non medical costs for 34 patients who were hospitalized were GH¢57.5 (US$38.33) for food and bed costs (Table 2).

**Table 2:** Mean cost per episode of meningitis per household (GH¢/US$).

Source of treatment	Number of cases	Treatment cost GH¢ (US$)	Transport cost GH¢ (US$)	Food & lodging cost GH¢ (US$)	Total cost GH¢ (US$)	Cost per case GH¢ (US$)
**Traditional Medicine**	8	25.5(17)	2.7(1.8)	25.0(16.7)	425.6(283.7)	5.5(3.7)
**Community health worker**	5	1.4(0.9)	2.0(1.3)	0.3(0.2)	18.5(12.3)	0.2(0.1)
**Clinic**	15	17.4(11.6)	2.9(1.9)	8.0(5.3)	424.5(283)	5.5(3.7)
**Hospital**	34	77.3 (51.5)	8.7(5.8)	57.5(38.3)	4862.0(3241.3)	63.1(42.1)
**Drug Shop**	15	3.3(2.2)	0.7(0.5)	2.0(1.3)	94.5(63)	1.2(0.8)
**Total direct cost**	77	124.6(83.1)	17.0(11.3)	93.1(62.1)	5825.1(3883.4)	75.7(50.5)

The indirect costs of GH¢76.85 (US$51.23) attributed to productivity losses was slightly higher than the direct medical care costs of GH¢75.70 (US$50.47) by 1.5%. Patients (26) with meningitis who were not students lost 29 days of productivity; while school going children lost 20 school days.

The household's average expenditure on both direct and indirect costs was GH¢152.55 (US$101.70) per meningitis case and was about 3 times higher than the average income range of GH¢50– GH¢100(US$33.33– US$66.67) reported by cases. Comparing the average expenditure of GH¢152.55 (US$101.70) on meningitis per household to the minimum wage of GH¢2.65(US$1.77) for Ghana in 2009, showed that a household needed 57.67 times its daily minimum wage or about two months minimum wage equivalent pay for treatment of a meningitis case.

Costs incurred by households for meningitis also varied depending on whether the household had insurance cover or not. Thirty-two (41.6%) of households with health insurance cover paid on average GH¢38.5 (US$25.67) per treatment while households without insurance cover 45 (58.4%) paid an average of GH¢177.9 (US$118.6) per case. This means that households without insurance cover paid approximately 4.6 times over the amount paid by the insured seeking care at the same hospital.

Thirty-three (41%) of the cases reported sequelae and fourteen of those cases (28.6%) incurred additional costs. Four sequelae cases sought care from a traditional healer and 10 cases returned to a doctor or nurse for further treatment. The average direct medical costs incurred by patients with sequelae seeing a doctor was GH¢2.1 (US$1.36) whereas it was as high as GH¢19.00 (US$12.67) for those who sought care from the traditional healer. The total direct medical and non-medical costs of sequelae were, on average, GH¢23.45 (US$15.63) per case (Table 3).

**Table 3:** Mean cost of sequelae (GH¢/US$).

Source of treatment	Number of cases	Treatment cost GH¢ (US$)	Transport cost GH¢ (US$)	Food cost GH¢ (US$)	Total cost (GH¢/US$)	Mean cos t GH¢ (US$)
**Nurse**	2	1.5(1)	2.0(1.3)	1.5(1)	10.0(6.7)	0.7(0.5)
**Doctor**	8	2.1(1.4)	9.8(6.5)	11.0(7.3)	183.4(122.3)	13.1(8.7)
**Traditional healer**	4	19.0(12.7)	6.7(4.5)	8.0(5.3)	134.8(89.9)	9.6(6.4)
**Total**	14	22.6(15.1)	18.5(12.3)	20.5(13.7)	328.2(218.8)	23.4(15.6)

In this study many more cases (24) came from the least poor households than from the poorest. However, socio-economic differences in health care were still observed between the least poor and poorest households. Whereas households of least poor paid GH¢125.00 (US$83.33) per meningitis case, the poorest household paid GH¢146.00 (US$97.33) per case (Table 4). The difference in the amounts paid was due to insurance cover as 23.7% (9) of those insured were from households of the least poor and 10.5% (4) were from the poorest households.

**Table 4:** Household Cost by socio-economic status (GH¢/US$).

Socio-economic status	Mean cost GH¢ (US$)	Confidence interval GH¢ (US$)
**Least poor**	125(83.3)	49.68953–200.4579(33.12635–133.6386)
**Less poor**	122 (81.3)	32.0054–211.7746(21.33693–141.1831)
**Poor**	98(65.3)	26.02775–170.7523(17.35183–113.8349)
**Very poor**	93(62)	47.15896–138.441(31.43931–92.294)
**Poorest**	146(97.3)	31.8737–187.9337 (21.24913–125.2891)

## Discussion

The average direct and indirect costs of treating meningitis in the Kassena-Nankana district was GH¢152.55 (US$101.7) per household, and up to GH¢175.55 (US$117.33) when sequelae occurred. Even without sequelae costs, this represents 57.67 times the daily minimum wage earned by Ghanaians in unskilled paid jobs representing about two months of lost minimum wage. In a district characterized by poverty and with over 70% of the population living below the poverty line [27], [28] this constitutes a large expenditure for households. Cases of chronic illness like sequelae in this study have been reported to lead to poverty and catastrophic health expenditures by households [5], [6].

A study in Burkina Faso on household cost of treating meningitis in Africa, reported households spending of US$90.00 per meningitis case and up to US$154.00 when meningitis sequelae occurred [29]. This is lower than the cost per meningitis case reported in this study. This difference could be due to the fact that our study was based on laboratory confirmed meningitis. A higher cost of treating laboratory confirmed meningitis (US$163) was reported in a study conducted in Vietnam [30].

The national health insurance scheme in Ghana provides free consultation, drugs and laboratory diagnosis for patients who are insured at a premium of Gh¢12.00 ($8.00) per member [31]. Meningitis cases that had insurance cover at the time of the illness spent less in direct medical costs than those who had no insurance cover. The direct cost per meningitis case represents over 6 times the insurance premium for informal sector workers from which most of the cases fall. Encouraging enrolment into the national health insurance system could substantially reduce costs of treating meningitis to the household and improve early seeking of care.

However, the indirect cost per meningitis case which is higher (50.38%) than direct costs in this study compares well with indirect costs reported by Colombini et al (2009) in Burkina Faso. Other cost of illness studies conducted in Ethiopia, Kenya and Ghana also reported indirect costs of care to be greater than direct costs [32]–[34]. Thus whereas payment mechanisms like health insurance could offset the direct costs of care to meningitis patients, indirect costs due to lost income resulting from ill-health remain a challenge.

The proportion of cases (62.5%) that reported to have previously vaccinated is high and this may not be related to the ineffectiveness of the vaccine. For instance, the MenA vaccine has been reported to be highly effective with no new cases of meningitis reported in those who have received one dose of the vaccine [35]. Nevertheless, increased incidences of meningitis has been observed in other studies conducted in the district and other parts of Ghana [13]. In this study vaccination against meningitis was based on self-reports over of a year which could suffer from recall bias. In addition, for some of the cases vaccinated, the vaccination may have occurred after the incident of meningitis.

Vaccination of population at risks which are often organized at the community level if provided at regular basis has the potential to prevent the disease and lead to reduction in indirect costs. The current targeting of meningitis vaccines to communities reporting outbreaks may not favour communities that may be at risk but do not report outbreaks. During meningitis outbreaks the convention is that vaccination campaigns target poorer communities. This study shows however that four times the number of meningitis cases came from the least poor than from the poorest quintile. This is surprising but it appears that cases from the wealthiest quintile may have a less perceived need for vaccination against meningitis due to their socio-economic status. This however places them in a vulnerable position and predisposes them to meningitis. Alternatively, health care providers may neglect vaccinating people living in affluent communities who are perceived to be more protected thus exposing them to meningitis.

Fewer cases in this study reported after effects of meningitis (40%) which is in contrast to high rates of after effects of meningitis reported elsewhere. For instance, an extensive review conducted by Peltola on studies conducted on meningitis revealed that about 50% of patients with meningitis developed long-term sequelae [36]. Differences in definitions of sequelae could also be the reasons in the variations in the rates of sequelae reported. Whereas subdural effusion was considered sequelae in some of these studies, only events occurring long after discharge from the hospital were referred to as sequelae in other studies [36]. It is possible that caregivers and patients themselves may not associate after effects that occur long after discharge from the health facility to the disease.

### Limitations of this study

Recall bias is a possible limitation. Some respondents were required to recall cost information over a period of four years (2008). It is possible that not all the respondents will accurately remember all the required information. Hence there is a possibility of either underestimation or over estimation of costs as respondents did not produce receipts on expenditure but only reported verbally. However, cost of hospitalization and health expenditure that has an impact on household resources are not easily forgotten. In addition, the probing mechanisms put in place during data collection minimized recalled problems and hence likely to reduce biases introduced in the study.

## Conclusion

Meningitis is a debilitating but preventable disease that affects people living in the Sahel and in poorer conditions. The disease poses an economic burden to households due to large out-of-pocket payments per case and cost of productive days lost to meningitis. Active vaccination of all household members saves households of GH¢175.95 (US$117.3) and impairment due to meningitis. Many meningitis cases are emerging from the richer households and vaccination should begin to target urban and affluent populations as they are at risk populations.
